# Determinants of Unresponsiveness to Treatment in Cutaneous Leishmaniasis: A Focus on Anthroponotic Form Due to *Leishmania tropica*

**DOI:** 10.3389/fmicb.2021.638957

**Published:** 2021-06-01

**Authors:** Mehdi Bamorovat, Iraj Sharifi, Razieh Tavakoli Oliaee, Abdollah Jafarzadeh, Ahmad Khosravi

**Affiliations:** ^1^Leishmaniasis Research Center, Kerman University of Medical Sciences, Kerman, Iran; ^2^Department of Immunology, Medical School, Kerman University of Medical Sciences, Kerman, Iran

**Keywords:** cutaneous leishmaniasis, environmental factors, poor treatment adherence, risk-factors, treatment unresponsiveness

## Abstract

Cutaneous leishmaniasis (CL) is a curable disease; however, due to various risk factors, unresponsiveness to CL treatments is inevitable. The treatment of CL has been firmly correlated with multiple determinants, such as demographical, clinical, and environmental factors, the host’s immune response, poor treatment adherence, the parasite’s genetic make-up, and *Leishmania* RNA virus. This study primarily focuses on the risk factors associated with different therapeutic outcomes following meglumine antimoniate (MA; Glucantime^®^) treatment and policy approaches to prevent unresponsiveness in CL patients with a focus on anthroponotic form (ACL). Findings suggest that effective preventive and therapeutic measures should be more vigorously implemented, particularly in endemic areas. Accordingly, extensive training is essential to monitor drug unresponsiveness regularly, especially in tropical regions where the disease is prevalent. Since humans are the fundamental reservoir host of ACL due to *L. tropica*, prompt detection, early diagnosis, and timely and effective treatment could help control this disease. Furthermore, major challenges and gaps remain: efficacious vaccine, new tools, and expert staff are crucial before CL can be definitively controlled.

## Introduction

Leishmaniasis is a multiplex disease induced by protozoa parasites belonging to over 20 different *Leishmania* species. It is a globally distributed disease, with an estimated prevalence rate of 12 million, an incidence of 0.5 million cases/year of the visceral form (VL), and 1.5–2.0 million cases per annum of the cutaneous form (CL). However, the current global burden is far beyond previously projected figures, especially in the main endemic areas of infection in Central and South America, North and East Africa, Southern Europe, the Middle East, and the Indian Subcontinent (Desjeux, 2004; Alvar et al., 2012; WHO, 2016; Bailey et al., 2017). According to the most recent estimate, more than one billion inhabitants in over 100 countries and territories are at risk of leishmaniasis: 431,850,220 people for CL and 616,220,219 people for VL (WHO, 2017). In 2017, seven countries (Afghanistan, Brazil, Colombia, Algeria, Iraq, Syrian Arab Republic, and Iran) represented more than 95% of new CL cases (WHO, 2017).

*Leishmania tropica* and *Leishmania major*, the two Old World cutaneous species, are the most prevalent etiological agents of CL in the tropics and semiarid subtropics, like the EMR region where 74% of the overall cases have been reported (Laffitte et al., 2005; Schwenkenbecher et al., 2006; Kumar et al., 2007; Bailey et al., 2017). Recent studies have indicated that CL has accelerated extraordinarily within Middle East conflict areas, such as Syria, Libya, Iraq, Afghanistan, and Iran (Sharifi et al., 2015; Bailey et al., 2017). In Iran, CL has a high prevalence in 18 of 31 provinces and is indicated as an endemic region for anthroponotic CL (ACL) due to *L. tropica* (Aflatoonian et al., 2014; Karimi et al., 2014; Sharifi et al., 2015). Reports on ACL date back decades, highlighting its presence in Iran as a classic and native form of the disease (Alvar et al., 2012). In urban locations at higher elevations, CL occurs due to *L*. *tropica*, existing within an anthroponotic cycle, which is primarily transmitted via the bite of the infected female phlebotomine sand fly, *Phlebotomus sergenti* (Yaghoobi-Ershadi, 2016). The disease affects a number of the poorest people in the world and is linked with population movement, poor housing conditions, deficient immune systems, and lack of monetary resources. Rural–urban labor migration, civil unrest, and climate change remain significant additional risk factors for ACL (Desjeux, 2001; Karimi et al., 2021). Leishmaniasis is also related to environmental modifications, including deforestation, dam building, urbanization, and irrigation schemes (WHO, 2017).

In Iran, meglumine antimoniate (MA, Glucantime^®^) has been applied as the drug of choice for the treatment of CL and VL cases, which has long been administered via the following methods: intra-lesionally alone, or along with cryotherapy, or systemically (Aflatoonian et al., 2019). Leishmaniasis is a curable disease (WHO, 2016); however, the emergence of unresponsive CL cases is inevitable due to various factors. As noted, the treatment of CL is also associated with several factors like environmental changes, the patient’s clinical status, the host’s immune response, and the parasite species (WHO, 2017; Bamorovat et al., 2018a). Therefore, the control of leishmaniasis needs multidisciplinary approaches.

**FIGURE 1:**
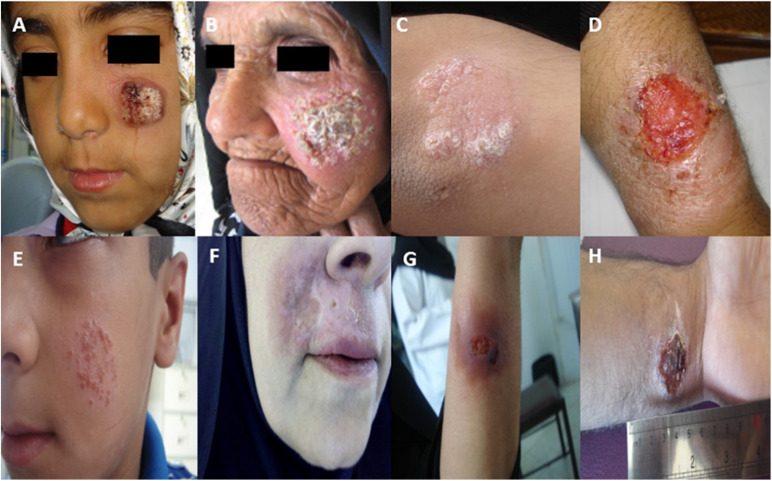
Active lesions from different unresponsive patients **(A–H)** with ACL due to *Leishmania tropica* in Kerman, southeast Iran [images obtained from Bamorovat et al. (2018a, 2019a,b), and Aflatoonian et al. (2019)].

An investigation performed by Dowlati (1996) showed that certain CL patients who are unresponsive to MA needed various treatment regimens (Dowlati, 1996). The rate of unresponsive (refractory) cases with ACL in some cities (such as Bam, Kerman and Mashhad) was reported to be 11–12% in Iran (Laffitte et al., 2005; Hadighi et al., 2006; Pourmohammadi et al., 2011; Karvar et al., 2016; Bamorovat et al., 2018a; Aflatoonian et al., 2019). Figure 1 shows active lesions from unresponsive patients with ACL due to *Leishmania tropica* in southeast Iran.

It should be noted that drug resistance in ACL, which is often transmitted anthroponotically, is more prevalent than in zoonotic CL (ZCL). Moreover, there is growing information that MA therapy can be a complicated process that is affected by variations in *Leishmania* species, susceptibility to treatment, differences in pharmacokinetics, and dissimilarities in host–drug immune system interactions. The lack of reference line data extracted from double-blind, randomized controlled clinical studies of available drugs for CL exacerbates the problem in terms of interpreting and describing treatment outcomes. The present review focused on the risk factors associated with different treatment outcomes following MA and thus formulates policy suggestions for the prevention of unresponsiveness in patients with ACL.

## Antileishmanial Medicines

### Pentavalent Antimonials (Gold Standard Treatment)

Two formulations of pentavalent antimonials are available: meglumine antimoniate (MA) and sodium stibogluconate.

In MA treatment, cases receive 20 mg/kg/day of MA intramuscularly (IM) for 21–28 days. Some of the cases receive intralesional (IL) administration of MA (20 mg/kg) every week for 12 weeks, along with biweekly liquid nitrogen cryotherapy (Shirzadi and Gouya, 2012). In some countries such as Iran, the treatment approaches are primarily based on the WHO protocol with minor modifications (WHO, 2010). The diameter of lesions ≥3 cm and/or number of lesions >5 or the lesion close to vital organs were the criteria for assigning the IM route of MA.

### Other Treatments

#### Amphotericin B Deoxycholate

Amphotericin B is a polyene antibiotic, and therapy must always be given in hospitals to monitor cases. A test dose of 1 mg, given by infusion, is recommended, followed by a total dose, 4–6 h later (WHO, 2010).

#### Lipid Formulations of Amphotericin B

Some formulations, such as liposomal amphotericin B, amphotericin B lipid complex, and amphotericin B colloidal dispersion, have been used to treat patients. These drugs are similar to amphotericin B deoxycholate in their cure rate, while their toxicity is negligible. They are given by intravenous (IV) infusion over 2 h (WHO, 2010).

#### Paromomycin

Paromomycin (aminosidine) is an aminoglycoside antibiotic, usually administered IM. The 15-mg/kg sulfate is equivalent to 11 mg/kg of the base, while the 20-mg/kg sulfate is equivalent to 15 mg/kg. Also, a topical formulation is available for CL (WHO, 2010).

#### Miltefosine

This alkyl phospholipid (hexadecylphosphocholine) was initially developed as an oral anticancer drug but was revealed to have an antileishmanial effect. Miltefosine should be taken after meals, and if multiple doses are going to be received, they should be given by divided dosages (WHO, 2010).

#### Ketoconazole, Fluconazole, and Itraconazole

These oral antifungal drugs have different efficacy in the treatment of leishmaniasis (WHO, 2010).

## Study Overview

The present review mainly focused on studies published in years from 1973 to 2021 on the risk determinants associated with CL and unresponsiveness to CL treatment globally and in Iran as well. For this purpose, a comprehensive literature review, encompassing research articles, reviews, and books from local and international databases, was conducted, using the following keywords: “*Leishmania tropica*,” “anthroponotic cutaneous leishmaniasis,” “cutaneous leishmaniasis” (CL), “risk-factors,” “unresponsive CL patients,” “drug resistance of leishmaniasis,” and “meglumine antimoniate.” It is worth mentioning that ZCL and ACL in Iran and the Eastern Mediterranean Region are endemic and notifiable diseases. However, there was no comprehensive report into the determinants of unresponsiveness to ACL treatment worldwide. In the current paper, the main risk-related factors for CL and ACL unresponsiveness were reviewed, described, and discussed.

## Risk-Related Factors for ACL Unresponsiveness

Based on literature, several risk factors may involve ACL unresponsiveness, including demographical factors, clinical factors, poor treatment adherence, environmental factors, the host immune responses, parasite genetic make-up, and *Leishmania* RNA virus. Each factor represents the main characteristics that cause unresponsiveness to standard drugs in ACL patients (Table 1).

**TABLE 1:** Risk-related factors in unresponsiveness to treatment in patients with cutaneous leishmaniasis.

**Risk related-factors**	**Potential profile**
Demographic factors	- Age; sex; education; job; marital status; nationality (Reithinger et al., 2010; Bamorovat et al., 2018a, 2021; Aflatoonian et al., 2019).
Clinical factors	- Location of the lesion; history of chronic diseases; the number of lesions; the size of the lesion; treatment course (complete or incomplete) (Bamorovat et al., 2018a, 2021; Aflatoonian et al., 2019)
Poor treatment adherence factors Patient-related factors	- Long distance from treatment setting; poor delivery of care education to family and caregivers; family support; poverty and low socioeconomic status; cultural and lay beliefs about illness and treatment; recent immigrant status; long duration of the lesion; adverse effects of treatment; absence of treatment for any reason; self-management of disease and treatment; unstable living conditions; forgetfulness; life stress; hopelessness and negative feelings; active participation in monitoring; belief of lack and poor effective drug and irregular treatment (Dunbar-Jacob and Mortimer-Stephens, 2001; WHO et al., 2003; DiMatteo, 2004)
Health services-related factors	- Lack of knowledge of health professionals about pain management; poor delivery of care education to the patient; the relationship between patient and health personnel; poorly developed health services; complex treatment regimens; inadequate treatment doses; a mistake in initial diagnosis; lack of clear instructions from health professionals; lack of knowledge and training for healthcare providers on managing chronic diseases (Dunbar-Jacob and Mortimer-Stephens, 2001; WHO et al., 2003; DiMatteo, 2004).
Environmental factors	- Building condition; wall condition; door and window net; interior housing condition; external toilet; the presence of small garden; hygienic dwelling condition; the presence of trees in the home; the presence of domestic animals (dog) in the home; the presence of manure in the home; lack of solid waste management; the presence of dogs in the region; the number of rooms and number of windows (Bamorovat et al., 2018a, 2021; Aflatoonian et al., 2019).
Host immune response factors	- Decreased expression of Th1 (IL-12 P40, IL-1β, TNF, IFN-γ) and increased expression of Th2 (IL-4, IL-10, IL-13, IL-9); other inflammation profiles involved in immune response (Bamorovat et al., 2019a; Oliaee et al., 2019).
The genetic make-up of parasite	
Genetic variation of parasite	- ITS1, Hsp70 and 7SLRNA genes (Bamorovat et al., 2018b)
Antimony resistance markers *Leishmania* RNA virus (LRV)	- ATP-binding cassette (ABC); multidrug resistance protein A (MRPA); aquaglyceroporin 1 (AQP1); c-glutamylcysteine synthetase (c-GCS); trypanothione reductase (TR); thiol-dependent reductase 1 (TDR1) and arsenate reductase (ACR2) markers (Oliaee et al., 2018). -Increasing production of pro-inflammatory cytokines and chemokines. It has been proposed as a potential cause of treatment failure (TF) in CL (Scott, 2011; Bourreau et al., 2015).

### Demographic Risk Factors

Household-level features have been shown as risk-related factors in patients with ACL. The supervision of specific household structures may help precise evaluations of household-level variations in populations at risk of ACL (Reithinger et al., 2010). An equal number of affected females and males were reported in community-based studies; however, studies based in healthcare centers revealed an apparent overrepresentation of males (Aflatoonian et al., 2019), most likely described by different healthcare-seeking behaviors for both sexes.

A study performed in Iran demonstrated that unresponsiveness to ACL treatment is significantly associated with increasing age. The impairment of innate cellular immunity (as caused by several factors, such as chronic diseases) may account for the increased unresponsiveness to treatment in older age ranges (Bamorovat et al., 2018a).

In a study evaluating treatment outcomes in ACL patients treated by Glucantime^®^, males (OR = 1.548) were demonstrated to be at higher risk of unresponsiveness to treatment (Aflatoonian et al., 2019). Male ACL patients frequently do not complete the standard medication, primarily because of negligible treatment adherence. This poor adherence to treatment might play a significant role in treatment failure (Aflatoonian et al., 2019). Marital status and occupation may affect treatment outcomes generally, but one study showed that these factors did not influence treatment outcomes (Bamorovat et al., 2018a).

### Clinical Risk Factors

Clinical factors, such as the number, location, and size of the lesions, treatment conditions, and concurrent chronic diseases, can affect the incidence and probable treatment outcomes of CL. Increased CL prevalence has been demonstrated in patients with long-lasting diseases such as diabetes and opium addiction; this finding has been attributed to defective innate immune responses. CL patients who have concurrent diabetes mellitus (DM) have exhibited greater infection frequency than those without DM. The increased prevalence of infections in patients with background diseases could be due to immune deficiencies (Sison et al., 1995; Geerlings and Hoepelman, 1999). It is also worth mentioning that opium use, which poses a significant public health concern, is a frequent phenomenon in the southeast part of Iran and its adjacent countries (Aflatoonian et al., 2014).

The results of a study performed in 2014 revealed that opium-addicted ACL patients developed more severe lesions in terms of number, size, and duration (Aflatoonian et al., 2014). However, the exact reasons for the difference in the severity of the CL lesions are not yet well understood. Based on studies conducted by Sacerdote et al. (1997) and Wei et al. (2003), opioids (including heroin and morphine, derivatives of opium alkaloids) may exert significant immunosuppressive effects (Sacerdote et al., 1997; Wei et al., 2003). Moreover, morphine has been demonstrated to disturb antibody production and cell-mediated immunity, as well as negatively interfering with the function of phagocytic cells, natural killer (NK) cells, reactive oxygen species (ROS), reactive nitrogen intermediates (RNI) (Yeager et al., 1995; Lysle and How, 2000; Bamorovat et al., 2018a). According to a study in Iran, diabetic patients and individuals with opium addiction, cardiovascular diseases, hypertension, and tuberculosis are more significantly prone to develop unresponsive forms of CL than those without a background of chronic disease or addiction (Bamorovat et al., 2018a,b).

In a multivariate regression analysis model, Aflatoonian et al. (2019) showed that facial lesions and multiple lesions were significant risk factors associated with unresponsiveness to ACL treatment in patients treated with MA (Aflatoonian et al., 2019). Among the various factors associated with treatment outcomes, multiple lesions may relate strongly to treatment unresponsiveness, compared with single lesions. Several studies have already recognized that each additional skin lesion will significantly increase the rate of treatment failure. Therefore, multiple lesions could result from a high parasite burden, which impairs *Leishmania* clearance (Valencia et al., 2012). As a consequence of multiple bites, there may be multiple lesions when a patient has encountered a nest of sandflies (Davies et al., 1995; Markle and Makhoul, 2004). Different genetic backgrounds with diverse susceptibility levels could presumably be introduced to the susceptible host in such a case. Different genotypes within the same species might react differently to treatment, as *L. tropica* represents several intraspecific genetic variants from the same area (Bamorovat et al., 2018b; Ghatee et al., 2018; Oliaee et al., 2018).

The exact mechanism which accounts for facial lesions being a major risk factor for treatment unresponsiveness (particularly in males) is not well understood. According to the results of similar studies, most lupoid leishmaniasis manifestations (leishmaniasis recidivans, LR) occur on the faces of male patients. This clinical presentation is one of the unresponsive ACL consequences due to *L. tropica* in the Old World (Sharifi et al., 2010, 2015). Most patients with LR lesions are highly resistant to MA (Esfandiarpour and Dabiri, 2007; Gitari et al., 2018; Aflatoonian et al., 2019).

### Poor Treatment Adherence Factors

Among other factors, poor treatment adherence is a major public health concern connected to developing resistance to treatment drugs. In developed countries, researchers have reported that treatment adherence among patients with underlying disease norms around 50% (Haynes et al., 2002). However, the amount and effect of poor treatment adherence are assumed to be even higher in developing countries, given the relative scarcity of health services and inequalities in healthcare access. Additionally, the effect of poor treatment adherence rises as the impact of chronic disease develops globally.

Many patients experience trouble in adhering to treatment regimes, with consequences including poor health outcomes and growing healthcare costs. Certain related factors concurrently predispose adherence. Patients’ capability to follow a treatment regimen appropriately is often conceded by more than one obstacle, typically associated with various factors, including socioeconomic factors, health personnel, the features of the specific disease, infection treatment, and patient-associated factors. Resolving the problems associated with each of these related factors is essential for patient treatments to be cured or improved (WHO et al., 2003).

The healthcare system has main potential to impact the treatment adherence behaviors of leishmaniasis patients. Systemic actions include improving the accessibility and availability of health services, providing education to leishmaniasis patients, data collection, monitoring and management, providing consultations to patients and healthcare services, making public support available to patients, and training courses in healthcare services (WHO et al., 2003).

In one study, it was found that patients with partial treatment or who had started treatment late (long-duration lesion) had greater odds of developing unresponsiveness than those who commenced treatment soon after disease onset. In the absence of active-case detection methods and common awareness, arbitrary treatments and inadequate medical services were all associated with late disease detection. Treatment started at least 4 months after disease onset was therefore shown to be more complex and challenging. Partial or irregular CL treatment occasionally occurs due to augmented drug resistance shown by *Leishmania* (Ouellette et al., 2004; Natera et al., 2007; Bamorovat et al., 2018a). In a multivariate regression analysis model, Aflatoonian et al. (2019) showed that indicators of poor treatment adherence (such as lesion duration of greater than 4 months) were major risk factors significantly associated with unresponsiveness to MA treatment among ACL patients (Aflatoonian et al., 2019).

Poor treatment adherence has a profoundly negative influence on the consequences of chemotherapy and treatment outcomes. The results of a study revealed that a substantial number of cases did not follow their physician’s advice. Thus, treatment adherence remains a major challenge for patients and healthcare providers (Aflatoonian et al., 2019). This remains a limiting factor in achieving optimal treatment outcomes.

### Environmental Risk Factors

Human health is significantly affected by environmental factors and housing conditions (Desjeux, 2001; Reithinger et al., 2010). Numerous factors have therefore contributed to the increased worldwide interest in basic and clinical research on CL. Cutaneous leishmaniasis, as a vector-borne disease, is able to adapt to environmental changes and continues to spread into urban and suburban residential areas of endemic countries. Household risk factors for the disease commonly include household design and building materials (quantity of floors and rooms, type of flooring), household existence of domestic animals, household proximity to recognized breeding areas for sandflies, and migration of household members (Demoulin and Renauld, 1998; Belkaid et al., 2002; O’Garra and Vieira, 2007; Ponte-Sucre et al., 2013; Bamorovat et al., 2018a, 2019a). Anthroponotic CL prevention and control programs should thus take into account the need for improvements and modifications in house construction, including wall smoothing and window screening (Sacerdote et al., 1997).

The question now is whether these associated-risk determinants (environmental factors) can play a role in leishmaniasis treatment response rates. In a study conducted in Iran, poor interior housing conditions were recognized as a major risk factor for ACL refractory forms. Recently, one study showed a significant relationship between poor interior housing conditions and the degree of unresponsiveness in ACL. The cracks and crevices in the floors, walls, and ceilings in low-quality housing can result in higher density of sandfly populations than more well-constructed houses. Considering the presence of parasites and the appropriate conditions for the proliferation and breeding of sandfly vectors, people in endemic areas who live in houses with poor interior conditions are thus more prone to CL infection (Chaves et al., 2013; Malaviya et al., 2014). Generally, individuals who live in unsuitable houses are from lower social classes, thus suffering from stressors such as finding employment, and often having difficulty in meeting their basic household needs. In case of CL infections, many people from such backgrounds start treatment too late or follow it partially and disrespect the consequences of infection. It appears that, due to the long period of the disease, as well as applying irregular or disordered treatment, the possibility of non-healing forms of CL developing increases for these groups (Bamorovat et al., 2018a).

Overall, environmental factors can influence disease incidence and probably ACL unresponsiveness to treatment. Nevertheless, comprehensive studies are needed to investigate each risk factor separately.

### Host Immune Responses

Immunity against leishmaniasis is facilitated through both innate (macrophages, dendritic cells, and neutrophils) and acquired (T lymphocytes) immunities, but the CD4^+^ T cell subset is essential for resistance (Jafarzadeh et al., 2019). Generally, cell-mediated immune response is the basis of solid immunity, while non-protective reactions are present as a robust humoral response in the lack of T cell-mediated immunity. It has been globally shown that the nature of the T cell-related response is a decisive parameter for drug resistance, despite the apparent response differences between mouse and human CL infections. The control of *Leishmania* infection is mainly established by T cell-mediated immune responses, which lead to the stimulation of immune cells, especially macrophages, which subsequently kill parasites. *In vivo* studies using leishmaniasis mouse models indicate that the Th1 and Th2 cell-related responses contribute to resistance or susceptibility, respectively (Jafarzadeh et al., 2019). It has been proven that protection against CL is closely associated with the growth of Th1 cell-linked immunity and IL-12/IFN-γ production. Primary experimental investigations have recognized a direct contrast between Th1 cell-mediated protection and Th2 cell-related susceptibility (Ponte-Sucre et al., 2012; Scott and Novais, 2016).

The Th1 cell-mediated pathway is associated with IFN-γ production, though it is not functionally heterogeneous. Recently, it has been demonstrated that a high-level, single specificity of IFN-γ-producing CD4^+^ T cells is not sufficient to overcome drug resistance to disease. The Th1 cell-mediated pathway launched by single-specificity CD4^+^ T cells [i.e., producing only IFN-γ or tumor necrosis factor-α (TNF-α)] has partial ability to expand into memory cells, in comparison to IL-2-producing ones. Therefore, their capability to provide durable protection is somewhat inadequate. Also, IFN-γ and TNF-α act synergistically in order to destroy parasites more efficiently (Jafarzadeh et al., 2019). Hence, multifunctional CD4^+^ T cells simultaneously produce various cytokines and presumably thus play a more significant role in reducing drug resistance to infection. The frequency of multifunctional CD4^+^ T cells (producers of IFN-γ, TNF-α, and IL-2) is associated with the extent of protection subsequent vaccination (Darrah et al., 2007). Findings show that the functional heterogeneity of Th1 cells to *Leishmania* parasites plays a vital role in infection resistance.

The Th2 cell-mediated humoral immune response may be induced during the infection, although these antibodies display no beneficial protection effects and are conversely related to non-responsive cases (Ponte-Sucre et al., 2012; Ponte-Sucre et al., 2017).

A set of cytokines and chemokines can influence the cellular immunity to leishmaniasis, comprising, but not limited to, IFN-γ, IL-4, IL-12, IL-13, TNF-α, and IL-10. The clinical outcomes of *Leishmania* infection correlate with the level and timing of cytokine generation. A range of immune cells has the potential to express these cytokines, such as CD4^+^ T cells (e.g., Th1- and Th2 cells), CD8^+^ T cells, double-negative T cells (CD4^–^ CD8^–^) (Gollob et al., 2008), DCs, macrophages, NK cells (Liese et al., 2008), and B cells (Ronet et al., 2010).

IL-12 and IFN-γ cytokines play a leading role in protection against different forms of leishmaniasis by inducing Th1 cell development. The IL-12/IFN-γ axis stimulates resistance by macrophage triggering nitric oxide (NO) production, which is essential for priming naive T cells toward the Th1 pathway (Jafarzadeh et al., 2019).

IFN-γ is the main cytokine involved in initiating the antileishmanial activities of macrophages through the induction of NO generation; it can stimulate macrophages alone or along with IL-7 or TNF-α (Gessner et al., 1993). TNF-α is a pro-inflammatory cytokine that is largely produced via triggered macrophages but is also generated by T cells, B cells, and fibroblasts. It contributes to resistance by controlling the replication of intracellular pathogens and by reducing the extent of the inflammatory responses. TNF-α can activate macrophages in a synergic manner with IFN-γ to express iNOS in their leishmanicidal activity (Liew et al., 1990).

It has been pointed out that IL-12 may be essential for ideal Th1 cell propagation and IFN-γ production, both of which are extensively enriched in the existence of IL-12 (which may also stimulate Th1 cell persistence) (Scott and Novais, 2016). Recently, investigations have shown that the fundamental memory CD4^+^ T cells, which are induced during *L. major* infections need IL-12 for IFN-γ production to differentiate into Th1 cells; in the absence of IL-12, these cells develop into IL-4 producer T cells (Pakpour et al., 2008). Moreover, genetic disturbances of the IL-12 gene result in the overexpression of harmful IL-4 responses and the development of advanced disease (Mattner et al., 1996). The majority of IL-12 is generated by antigen-presenting cells (APCs), such as DCs, macrophages, and neutrophils (von Stebut and Udey, 2004); however, *L. major* can block its production in macrophages (Zhang et al., 2013) selectively. Therefore, DCs seem to be the primary source of IL-12 in antileishmanial action, along with DC-derived IL-1α/β to potentiate Th1 cell expansion and develop resistance to CL (von Stebut et al., 2003).

IL-4 is considered as a Th2 cell-related pathway in leishmaniasis. Th2 cell response is driven by IL-4, which promotes vulnerability through macrophage inhibition and the reduction of IL-12 expression. IL-13 shares many features with IL-4, as both have a mutual signaling pathway using the IL-4 receptor alpha (Mohrs et al., 2000). IL-13 exerts disease-elevating features and acts independently of IL-4 (Matthews et al., 2000), indicating the additive effects of IL-13 and IL-4. High amounts of IL-13 inhibit the Th1 cell-mediated responses via preventing IL-12 generation by macrophages, inducing a skewing toward harmful Th2 cells.

In leishmaniasis, IL-10 (as a potent immunosuppressive cytokine) is vital for the persistence of the parasite (Belkaid et al., 2001) and can exacerbate the disease (Belkaid et al., 2002; Scott and Novais, 2016). It is also an effective suppressor of macrophage stimulation and prevents the maturation of DCs (O’Garra and Vieira, 2007). IL-10 is mainly generated by Treg cells, Th2 cells, and M2 macrophages during leishmaniasis (Jafarzadeh et al., 2019). IL-9 has also been revealed to be involved in disease susceptibility. It is prominently generated by Th2- and Th9 cells (Demoulin and Renauld, 1998), and its generation can be either self-governing or IL-4-dependent (Ponte-Sucre et al., 2013).

Recently, Bamorovat et al. (2019a) demonstrated that the expression profiles of IL-1β, IL-12, P40, and IFN-γ genes in *L. tropica*-motivated and non-motivated cells in unresponsive patients were significantly lower than those in responsive patients. Conversely, IL-10 and IL-4 gene expression profiles were increased in unresponsive patients compared to responsive ones. Possibly, these diverse immune responses resulting from several effector cells play a vital role in the pathology and progress of unresponsive ACL forms.

An upregulation of IL-27 expression also occurs after *L. major* infection, and the infected DCs have been suggested as being the primary producers of IL-27 through the initial phase of CL (Zahn et al., 2005; Anderson et al., 2009).

Specific pattern recognition receptors, especially TLRs, may play a decisive role in leishmaniasis treatment outcomes (Jafarzadeh et al., 2019). Both anti-*Leishmania* and pro-*Leishmania* activities have been attributed to TLR2, as a receptor of parasite-related LPG (Jafarzadeh et al., 2019). It has been observed that TLR2^–/–^ C57BL/6 mice exhibit more extensive lesions and produce elevated amounts of specific anti-parasite IgG1 (Halliday et al., 2016). These findings suggest that the TLR2 defect causes disease exacerbation and parasite growth through the enhanced Th2 cell-related responses. However, it has been indicated that the neutrophils of unresponsive CL patients expressed higher levels of TLR2, TLR4, and TLR9 compared to the neutrophils of asymptomatic patients (leishmanin skin-test positive) or healthy individuals (Halliday et al., 2016; Oliaee et al., 2018, 2019). However, the TLR2-related impacts can be influenced by parasite species, ligand density, and the signals coming from other PRRs (Jafarzadeh et al., 2019).

**FIGURE 2:**
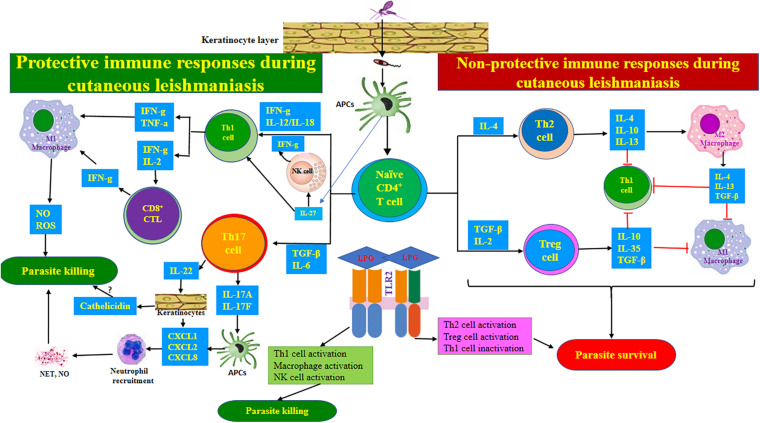
Immune responses during cutaneous leishmaniasis. After injecting the parasite into the skin, the *Leishmania*-derived antigens are offered to naïve CD4^+^ T-lymphocytes by macrophages. Then, naïve CD4^+^ T-lymphocytes are differentiated to the various subsets of effector T cells according to specific local cytokines in the milieu. The Th1 and CTL-related immune responses exert protective impacts, while Th2- and Treg cell-associated responses support parasite survival. Th17 cell-mediated responses may be protective through neutrophil recruitment. IL-27 and IL-23 can contribute to protection via supporting Th1 and Th17 cell responses, respectively. TLR2 recognizes parasite-derived LPG and exhibits functional duality during CL. The anti-*L. major* impacts of TLR2 can be exerted mainly by activating Th1 cells, macrophages, and NK cells, while pro-*L. major* impacts may be mediated by inactivating Th1 cells while activating Th2 and Treg cells.

Even though *Leishmania* activates both innate and adaptive immunity, it has advanced various mechanisms to interfere with the development of the immune system and overcome the host’s resistance. Comprehensive knowledge of anti-parasitic host immune responses, in addition to the production and maintenance of anti-*Leishmania* immunological memory, provides confidence for the expansion of novel strategies allowing for effective vaccines, immunotherapies, and chemotherapies. A well-balanced immunological response to *Leishmania* infections is necessary to control parasite replication while avoiding the expansion of pathology. There is an increasing tendency to expand immunotherapies that develop the effectiveness of existing leishmanicidal drugs by reinstating the host’s immune mechanism. Therefore, a better understanding of the role of the host’s immune response in the resistance to leishmaniasis can offer novel insights to design new strategies for therapeutic interventions. Figure 2 lays out the immune responses during CL.

### Genetic Make-Up of Parasite

#### Parasite Genetic Variations

Lack of treatment response can be related to several aspects; one of them is the parasite’s genetics. Over 20 pathogenic *Leishmania* species have been recorded, with 20 known hosts so far (Claborn, 2014).

Two principal Old World cutaneous species, *L. major* and *L. tropica*, are the widespread etiologic agents of CL in the tropics and subtropics, particularly in Middle Eastern countries (Laffitte et al., 2005; Schwenkenbecher et al., 2006; Kumar et al., 2007).

Three genes have been extensively addressed in the systematic literature as discriminating *Leishmania* isolates (Garcia et al., 2004; Fraga et al., 2012; Montalvo et al., 2012; van der Auwera et al., 2013), namely, the internal transcribed spacer (ITS1) of ribosomal DNA (rDNA) (El Tai et al., 2000; Nasereddin et al., 2008; Talmi-Frank et al., 2010; Odiwuor et al., 2011), the heat shock protein 70 (Hsp70), and the 7SL RNA (Zelazny et al., 2005; Stevenson et al., 2010). Gene sequence analysis is the standard method for routine practice in medical, molecular, and epidemiological investigations on a universal scale (van der Auwera et al., 2014). Furthermore, the molecular characterization of *Leishmania* samples is a suitable method for the assessment and selection of an appropriate treatment regimen. Certain forms of leishmaniasis could be related to specific species of *Leishmania*, which may affect initial identification and treatment outcomes; hence, molecular documentation and classification should not be restricted to simple species identification. Therefore, the significance of intraspecies diversity in the progression of unresponsive and responsive ACL forms is undeniable (Bamorovat et al., 2018b).

A major question concerning leishmaniasis is whether the genetic composition of the organism is responsible for the treatment outcome or not. A study performed in the Kerman and Bam regions of Iran revealed that distinctive genetic heterogeneity was closely linked to the ITS1 region of *L. tropica* isolates, obtained from unresponsive and responsive cases; these nucleotide differences were not identified in other *L. tropica* isolates. This diversity may demonstrate intraspecies difference within the *L. tropica* isolates. Molecular variations in various ACL patients caused by *L. tropica* have been described frequently within several endemic areas, particularly in *Leishmania* complex (Mebrahtu et al., 1992; Schönian et al., 2001). Extensive arrays of polymorphisms within most *Leishmania* species, especially in the ITS1 region, have been recognized as being associated with various clinical presentations (Rodriguez-Bonfante et al., 2003; de Oliveira et al., 2007). According to the results of studies on the nucleotide variation of the ITS1 region, such polymorphism in the *L. tropica* complex was not beyond expectations (Ghatee et al., 2013, 2014).

The phylogenetic analysis appears to have different abilities in terms of detecting *Leishmania* species. In a study on responsive and unresponsive patients with leishmaniasis, Bamorovat et al. (2018a, b) reported polymorphisms in the ITS1 region of the isolates but did not detect discordant nucleotides in the Hsp70 and 7SL RNA genes (Bamorovat et al., 2018b). Since some single-nucleotide differences were shown in isolates from both unresponsive and responsive patients, possible genomic dissimilarities between the parasitic species of the unresponsive and responsive cases may be assumed. However, while single-gene sequencing (as the most suitable method in terms of its clinical forms) can identify *Leishmania* at the species and intraspecies levels, it does not help characterize the strain of *Leishmania* complex by single-locus genes (van der Auwera and Dujardin, 2015). Therefore, additional target genes and high-resolution methods are highly suggested for a more comprehensive investigation into the various clinical features of ACL (Boité et al., 2012; Zhang et al., 2013; Montalvo et al., 2014; van der Auwera and Dujardin, 2015).

Appropriate characterization of *Leishmania* species is vital for accurate identification and correct prediction of the disease and decision-making concerning therapy and management procedures (Ponte-Sucre et al., 2013).

Molecular and biochemical differences between various species offer construction for phylogenetic examination and improve species characterization (Cupolillo et al., 2000; Lewin et al., 2002). Regarding the noticeable molecular and biochemical differences among various species, variations in the intrinsic sensitivity of unlike *Leishmania* species to treatment is not surprising (Croft et al., 2006).

#### Antimony Resistance Markers

To be active against the *Leishmania* genus and achieve effective treatment performance, pentavalent antimonies (SbV) like MA need to arrive the host macrophages, pass the phagolysosomal membrane, and combat intra-macrophage amastigotes (Leishman bodies). Moreover, SbV, as a prodrug, needs likely be transformed to a trivalent antimonial (SbIII) form to be active (Ouellette et al., 2004). The activation mechanism of SbV is not well identified, and the specific place of this transformation is uncertain. One investigation revealed that SbV is reduced to SbIII by axenic amastigotes, but not promastigotes (Shaked-Mishan et al., 2001). However, other documents have proposed that the reduction most likely occurs within the macrophage cells rather than in the parasites (Roberts and Rainey, 1993; Sereno et al., 1998). These findings are not necessarily inconsistent, and the reduction of SbV to SbIII possibly takes place both in the parasite and in the host cells. In addition to the direct influence of SbV on the parasite, it may affect cell signaling, which might consequently lead to cell death (Ouellette et al., 2004).

Various genes are involved in inducing parasite-resistant forms. The main ones include aquaglyceroporin 1 (AQP1), multidrug resistance protein A (MRPA), γ-glutamylcysteine synthetase (γ-GCS), trypanothione reductase (TR), and thiol-dependent reductase 1 (TDR1).

Gene deletion or minor expression of AQP1, a membrane transporter of SbIII, can render the parasite into a resistant form (Marquis et al., 2005; Mandal et al., 2010). It has been found that elevated levels of MRPA, as an ATP-binding cassette (ABC) transporter, affect the efflux of drugs in intracellular vesicles by sequestering thiol-metal complexes (Ponte-Sucre et al., 2017). Increased expression of thiol biosynthetic enzymes, such as TR and γ-GCS, and decreased levels of TDR1, as a reducer enzyme of SbV, are also involved in drug resistance (Ouellette et al., 2004; Croft et al., 2006; Jeddi et al., 2011). It has been observed that in most species, lower expression of the AQP1 gene was represented in resistant isolates more than in sensitive ones (Kumar et al., 2012; Oliaee et al., 2018). Resistant isolates also showed an upregulation of the MRPA and γ-GCS genes (Kazemi-Rad et al., 2013; Aflatoonian et al., 2016).

SbV enters the amastigotes through an unknown transporter, and SbIII passes the membrane via AQP1. The conversion of SbV into SbIII can take place within the parasite by both the non-enzymatic reduction of glutathione (GSH) and trypanothione (TSH) and by the enzymatic reduction of TDR1 and arsenate reductase (ACR2) (Ouellette et al., 2004).

TDR1, a parasite-specified enzyme that includes domains with resemblances to omega glutathione transferase, has been revealed to act as a catalyst in the conversion of SbV into SbIII, using glutathione as a reductant. The higher expression of this enzyme may cause the amastigotes to become more susceptible to antimonials (Denton et al., 2004). This increase in TDR expression has been indicated as being 3.55-fold higher in ACL-responsive patients versus unresponsive ones (Oliaee et al., 2018).

Often, an upregulation of the enzymes involved in the biosynthesis of GSH (gamma-glutamylcysteine synthetase—γ-GCS) and ornithine and polyamines (ornithine decarboxylase—ODC) causes high levels of trypanothione in unresponsive strains (Haimeur et al., 1999). SbIII is likely to interact with a number of cellular targets but may also cause conjugation with several thiols, comprising cysteine, trypanothione, and glutathione. It is uncertain whether the conjugation is enzymatically mediated or not. Increased trypanothione levels have been often shown in antimony-resistant isolates, which presumptively increase conjugation to the metal form. There are two likely scenarios for the metal-thiol complex: it can be either confiscated into an organelle by the MRPA ABC transporter or thrown out of the cell by an efflux pump, which probably corresponds to another ABC transporter (Ouellette et al., 2004).

The ABC proteins represent the most prominent family of transmembrane proteins identified to date and can be found in all prokaryote and eukaryote cells (Higgins, 1992). According to Saurin et al. (1999), ABC proteins function differently within eukaryotes and prokaryotes. In eukaryotes, they are involved in export actions, while in prokaryotes, they play a role in both export and import actions. These proteins are primarily liable for transportation through cellular membranes in a range of macromolecules like peptides, polysaccharides, lipids, and polypeptides. Furthermore, ABC proteins may also have a role in biological activities such as DNA or even gene expression beyond simply molecule transportation (Sauvage et al., 2009). ABC proteins were first identified and documented in *Leishmania* during drug resistance studies, showing that these proteins can play analogous roles for other parasitic protozoa (Klokouzas et al., 2003) and cancer cells (Gottesman et al., 2002). ABCA, ABCB, and ABCC to ABCH are a number of the subfamily members of the ABC transporters.

*Leishmania* species have a complete set of ABC proteins with a structural variation visualized by the genome database. Studies on the role of ABC transporters in the various aspects of leishmaniasis are limited. Some ABCA subfamily members are responsible for transporting various phospholipids, while the other members of ABCB, ABCC, and ABCG are related to drug resistance (Leprohon et al., 2006), a significant challenge in the chemotherapy of diseases. Developing a proper understanding of ABC transporter proteins and their role in *Leishmania* is crucial for detecting the mechanisms involved in related drug resistance. It is essential to know whether the ABC transporter proteins (and their metamorphoses at the molecular level among different *Leishmania* species) are related to the mode of actions of drug resistance. It is also worth mentioning that there is a different innate and intrinsic sensitivity to SbV in various *Leishmania* species (Croft et al., 2006; Ponte-Sucre et al., 2013).

Rapid efflux is a mechanism of drug resistance responsible for exporting drugs out of the cell and lowering their concentrations, which are conducted by ABC transporters. The major transporters in leishmanial species are MRP and P-glycoprotein (P-gp). P-gp-type pumps have been identified as being involved in the export and efflux of anionic compounds in conjugation with thiols. MRPA was the first characterized ABC protein. This protein is not responsible for refluxing drugs from plasma membranes but is responsible for sequestering thiol-metal conjugates into an intracellular vesicle and inducing resistant forms (Sundar and Goyal, 2007). In different studies, the overexpression of MRPA markers was revealed in the unresponsive isolates of *L. donovani* and *L. tropica* species (Kumar et al., 2012; Oliaee et al., 2018).

Vanadate-sensitive P-type ATPases, including P-gp (which is involved in MDR in eukaryote cells), were illustrated to be significantly increased in the plasma membrane of methotrexate-resistant *L. tropica* and arsenite-resistant *L. donovani* experimental mutants. Currently, the functionality of verapamil-sensitive P-type efflux pumps has been shown in *L. donovani* species resistant to antimony. The efficient measurement of efflux pumps further confirmed these results.

**FIGURE 3:**
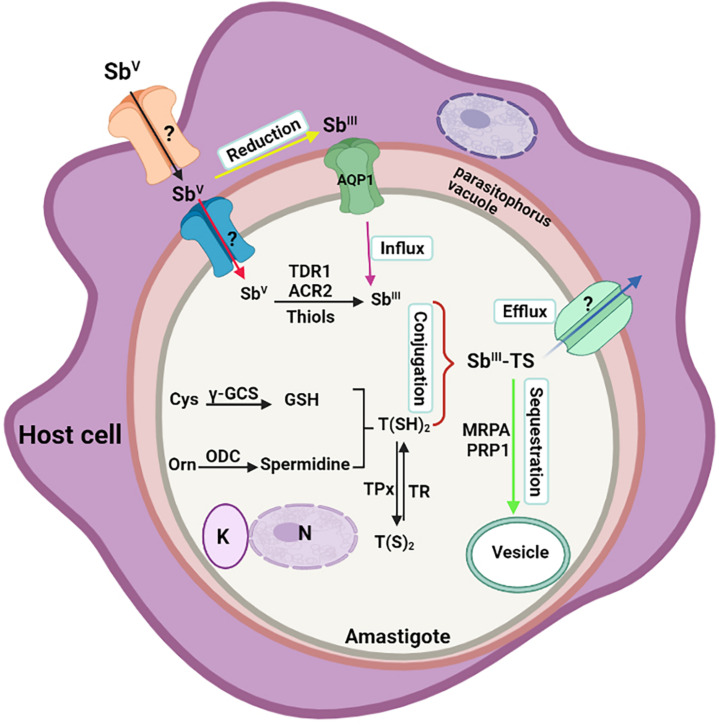
Schematic diagram of molecular mechanisms involved in antimony resistance in *Leishmania*. The Figure illustrates a Leishman body inside a parasitophorous vacuole of the host macrophage. It represents the influx and reduction of antimonials to enter the parasite and other parasite mechanisms to reduce the effectiveness of the drug, causing the resistance form. AQP, aquaporin; MRPA, multidrug resistance protein A; TR, trypanothione reductase; γ-GCS, glutamylcysteine synthetase; TDR1, thiol-dependent reductase 1; ACR2, arsenate reductase 2; ODC, ornithine decarboxylase; TPx, thioredoxin peroxidase; PRP1, pentamidine resistance protein 1; N, nucleus; K, kinetoplast.

In conclusion, antimonial resistance is a multifactorial phenomenon. Some of the primary mechanisms for resistance to antimony include reduced drug uptake, elevated intracellular levels of thiol, sequestration, and rapid drug efflux. Based on the outcomes of a recent study from Nepal, variations in genetic make-up were proposed as a significant actuation force in the progress of diverse sodium stibogluconate-resistant (SSG-R) phenotypes. The parasite’s intrinsic capacity, which is determined by its genetics, is supposed to vary among distinct genetic populations (Adak and Datta, 2015). Loss of drug stimulation or reduced activation could be one of the mechanisms involved in the appearance of resistant parasites (Ouellette et al., 2004). Figure 3 shows a representative diagram of the molecular modes of action involved in antimony resistance in CL.

### *Leishmania* RNA Virus

*Leishmania* RNA virus (LRV), as a member of the *Totiviridae* family, is a double-stranded RNA (dsRNA) of about 5.3 kb that encodes a non-enveloped capsid and an RNA-dependent RNA polymerase. Various species of the animal kingdom can be infected by this family, including arthropods, fish, bats, yeast, molds (*Helminthosporium* spp., and *Aspergillus*), as well as different human pathogens (*Trichomonas vaginalis* and *Giardia lamblia*).

Up to now, LRV-1 has been identified from different *Leishmania* spp., especially in the New World species of *L. (Viannia) braziliensis*, *L. V. guyanensis*, *L. V. panamensis*, *L. amazonensis*, and *L. V. lainsoni*, which are responsible for occurring of disseminated, diffuse, or mucocutaneous forms. Additionally, the virus was also detected in the Old World species of *L. aethiopica*, *L. major*, and *L. infantum* (Bourreau et al., 2015; Hajjaran et al., 2016; Saberi et al., 2019).

The virus increases immune responses toward production of pro-inflammatory cytokines and chemokines of TNF-α, IL-6, CXCL10, and CCL5, which is mediated by binding of viral RNA to TLR3, an RNA receptor expressed within the parasited endosomal vesicle (Scott, 2011), although in a study on *L. V. panamensis* infection, no increase was identified in the expression of pro-inflammatory markers by LRV-1 (Kariyawasam, 2020).

It has been mentioned that LRV can be proposed as a potential cause of treatment failure (TF) in CL (Abtahi et al., 2020), as in *L. guyanensis* infection, all patients infected with LRV1-negative *L. guyanensis* were treated after one or two doses of pentamidine, but 27.3% of LRV1-positive patients represented persistent infection and symptomatic relapse that needed additional treatment or using of second-line drugs (Bourreau et al., 2015).

In mucocutaneous forms, high burden of LRV-1 was detected in metastasizing parasites that increased parasite persistence and footpad swelling in mice (Ives et al., 2011). Also, LRV-1 was significantly associated with a high risk of treatment failure in *L. braziliensis* patients of Peru and Bolivia (Adaui et al., 2016). Although Abtahi et al. (2020) showed no LRV-1 in any of the L. major treatment failure (TF) samples, seven treatment responsive (TR) isolates and two TF isolates were positive for LRV-2, that no significant difference was identified among LRV-2 and response to Glucantime^®^.

In addition, Ginouvès et al. (2020) study, the presence or genotypes of LRV-1 in American tegumentary leishmaniasis (ATL) patients with *L. guyanensis* did not correlate with pentamidine treatment failure.

## Discussion

Protective, prophylactic, and therapeutic strategies focusing on endemic areas should be more strongly pursued. Accordingly, more training is required to monitor drug unresponsiveness regularly, especially in tropical regions where the disease is present. The improvement of diagnostic approaches with high sensitivity and specificity levels would provide a significant improvement in the management of leishmaniasis. The most important action to improve disease status in endemic areas is to develop health infrastructure at a peripheral level. Medical education programs and training are vital to maximizing currently effective *Leishmania* prevention and control programs. Moreover, public communities should be educated to be able to engage in healthcare processes and treatments and take informed actions. The appropriate management of concurrent chronic conditions and coinfections, such as diabetes, is also essential.

Free drugs offered via suitable medical practices and an efficient health system are required. Increasing unresponsiveness makes disease management difficult; despite advances in chemotherapeutic options, promoting research into vaccine development is another crucial need. Chemotherapy alone would be unlikely to eliminate the disease. Enhancing the host immune response by vaccination, or protecting immunodeficient patients from being bitten by the phlebotomine sandflies, would provide a unique approach to disrupt the life cycle of the etiological agent.

Supplementary immunomodulatory mediators with exact treatments avert the opposing immunological deviations that occur throughout CL infection. Immunomodulators remain prescribed once wounds are resulting from immunocomplex deposition (Alvar et al., 2012).

Improved drug resistance monitoring methods are required to observe and investigate the phenotypic susceptibility of parasite isolates and the molecular changes causing modifications in either the drug target or modes of action, which modify intra-parasite levels through the application of an active drug.

A suitable array of medicines with different targets and no cross-resistance could be used to avoid drug resistance; however, introducing new targets and new drugs may provide better results. To control the disease, chemotherapy is therefore the only measure needed at present. The development of superior and more efficient ACL treatment drugs is highly required to diminish infections in endemic areas (Bamorovat et al., 2019b).

Combination drug therapies, which have been shown to be a crucial feature of antimicrobial therapy, can provide several benefits. Combinations with synergistic, potentiating, or additive activities may prevent the appearance of drug resistance, minimize the doses needed, reduce lethal adverse effects, and promote the range of anti-disease activity. For instance, a leishmanicidal drug with either anti-inflammatory or immunomodulatory effects could be efficient in the treatment of CL. A previous study on combination therapy for ACL indicated that combining the first-line and second-line drugs could improve their efficacy (Croft et al., 2006; Bamorovat et al., 2019b).

## Conclusion

Limited treatment options are available for ACL. Additionally, the emergence of drug resistance because of the anthroponotic nature of the organism is further confounding leishmaniasis control. Regular monitoring of drug resistance and identifying determinants associated with unresponsive forms of ACL are essential practices to develop preventive measures and coping strategies. It is necessary to discover the mode of action of the medications involved in resistance to design a superior and more effective drug schedule against ACL. In the absence of realistic vector and reservoir control approaches and efficacious vaccines against leishmaniasis, using effective chemotherapy remains the therapy’s pillar. At present, since the armory of antileishmanial drugs is limited, strategies should be designed for monitoring and preventing medicine resistance. Therefore, early detection, prompt diagnosis, and effective treatment could play a crucial role in preventing unresponsive patients.

## Author Contributions

MB and IS contributed to the conception and design of the study. MB organized the database. MB, IS, and RT wrote the first draft of the manuscript. MB, IS, RT, AJ, and AK contributed to the manuscript revision, read, and approved the submitted version.

## Conflict of Interest

The authors declare that the research was conducted in the absence of any commercial or financial relationships that could be construed as a potential conflict of interest.
